# The LiberAction Project: Implementation of a Pediatric Liberation Bundle to Screen Delirium, Reduce Benzodiazepine Sedation, and Provide Early Mobilization in a Human Resource-Limited Pediatric Intensive Care Unit

**DOI:** 10.3389/fped.2021.788997

**Published:** 2021-12-08

**Authors:** Matteo Di Nardo, Francesca Boldrini, Francesca Broccati, Federica Cancani, Tiziana Satta, Francesca Stoppa, Leonardo Genuini, Giorgio Zampini, Salvatore Perdichizzi, Gabriella Bottari, Maximilian Fischer, Orsola Gawronski, Annamaria Bonetti, Irene Piermarini, Veronica Recchiuti, Paola Leone, Angela Rossi, Paola Tabarini, Daniele Biasucci, Alberto Villani, Massimiliano Raponi, Corrado Cecchetti, Karen Choong

**Affiliations:** ^1^PICU, Children's Hospital Bambino Gesù, Istituto di Ricovero e Cura a Carattere Scientifico (IRCCS), Rome, Italy; ^2^Unit of Clinical Psychology, Department of Neurological Sciences, Children's Hospital Bambino Gesù, Istituto di Ricovero e Cura a Carattere Scientifico (IRCCS), Rome, Italy; ^3^Pediatric Emergency Unit, Department of Medical and Surgical Sciences (DIMEC), St. Orsola-Malpighi Hospital, University of Bologna, Bologna, Italy; ^4^Professional Development, Continuing Education and Research Unit, Medical Directorate, Bambino Gesù Children's Hospital, Istituto di Ricovero e Cura a Carattere Scientifico (IRCCS), Rome, Italy; ^5^Functional Rehab Unit, Neurorehabilitation and Robotics Department, Bambino Gesù Children's Hospital, Istituto di Ricovero e Cura a Carattere Scientifico (IRCCS), Rome, Italy; ^6^Respiratory Physiotherapy, Pediatric Pulmonology and Respiratory Intermediate Care Unit, Sleep and Long-Term Ventilation Unit, Academic Department of Pediatrics (DPUO), Bambino Gesù Children's Hospital, Istituto di Ricovero e Cura a Carattere Scientifico (IRCCS), Rome, Italy; ^7^Department of Emergency, Intensive Care Medicine and Anesthesiology, Fondazione Policlinico Universitario “A. Gemelli” Istituto di Ricovero e Cura a Carattere Scientifico (IRCCS), Rome, Italy; ^8^Medical Directorate, Bambino Gesu' Children's Hospital, Istituto di Ricovero e Cura a Carattere Scientifico (IRCCS), Rome, Italy; ^9^Department of Pediatrics, McMaster University, Hamilton, ON, Canada; ^10^Department of Health Research Methods, Evidence and Impact, McMaster University, Hamilton, ON, Canada

**Keywords:** delirium, bundle, pediatric intensive care, benzodiazepine (BDZ), early mobilization, sedation

## Abstract

**Background:** Delirium, bed immobilization, and heavy sedation are among the major contributors of pediatric post-intensive care syndrome. Recently, the Society of Critical Care Medicine has proposed the implementation of daily interventions to minimize the incidence of these morbidities and optimize children functional outcomes and quality of life. Unfortunately, these interventions require important clinical and economical efforts which prevent their use in many pediatric intensive care units (PICU).

**Aim:** First, to evaluate the feasibility and safety of a PICU bundle implementation prioritizing delirium screening and treatment, early mobilization (<72 h from PICU admission) and benzodiazepine-limited sedation in a human resource-limited PICU. Second, to evaluate the incidence of delirium and describe the early mobilization practices and sedative drugs used during the pre- and post-implementation periods. Third, to describe the barriers and adverse events encountered during early mobilization.

**Methods:** This observational study was structured in a pre- (15th November 2019–30th June 2020) and post-implementation period (1st July 2020–31st December 2020). All patients admitted in PICU for more than 72 h during the pre and post-implementation period were included in the study. Patients were excluded if early mobilization was contraindicated. During the pre-implementation period, a rehabilitation program including delirium screening and treatment, early mobilization and benzodiazepine-sparing sedation guidelines was developed and all PICU staff trained. During the post-implementation period, delirium screening with the Connell Assessment of Pediatric Delirium scale was implemented at bedside. Early mobilization was performed using a structured tiered protocol and a new sedation protocol, limiting the use of benzodiazepine, was adopted.

**Results:** Two hundred and twenty-five children were enrolled in the study, 137 in the pre-implementation period and 88 in the post-implementation period. Adherence to delirium screening, benzodiazepine-limited sedation and early mobilization was 90.9, 81.1, and 70.4%, respectively. Incidence of delirium was 23% in the post-implementation period. The median cumulative dose of benzodiazepines corrected for the total number of sedation days (mg/kg/sedation days) was significantly lower in the post-implementation period compared with the pre-implementation period: [0.83 (IQR: 0.53–1.31) vs. 0.74 (IQR: 0.55–1.16), *p* = 0.0001]. The median cumulative doses of fentanyl, remifentanil, and morphine corrected for the total number of sedation days were lower in the post-implementation period, but these differences were not significant. The median number of mobilizations per patient and the duration of each mobilization significantly increased in the post-implementation period [3.00 (IQR: 2.0–4.0) vs. 7.00 (IQR: 3.0–12.0); *p* = 0.004 and 4 min (IQR: 3.50–4.50) vs. 5.50 min (IQR: 5.25–6.5); *p* < 0.0001, respectively]. Barriers to early mobilization were: disease severity and bed rest orders (55%), lack of physicians' order (20%), lack of human resources (20%), and lack of adequate devices for patient mobilization (5%). No adverse events related to early mobilization were reported in both periods. Duration of mechanical ventilation and PICU length of stay was significantly lower in the post-implementation period as well as the occurrence of iatrogenic withdrawal syndrome.

**Conclusion:** This study showed that the implementation of a PICU liberation bundle prioritizing delirium screening and treatment, benzodiazepine-limited sedation and early mobilization was feasible and safe even in a human resource-limited PICU. Further pediatric studies are needed to evaluate the clinical impact of delirium, benzodiazepine-limited sedation and early mobilization protocols on patients' long-term functional outcomes and on hospital finances.

## Introduction

Advancements in the management of critically ill children have led to a considerable improvement in patient survival (1–3), however, this has been accompanied by an increase of physical, cognitive, and psychosocial morbidities leading to delayed recovery, functional impairments, and reduced quality of life. These comorbidities, which constitute the base for the development of the pediatric post-intensive care syndrome (p-PICS) (4, 5), are often caused by the use of high levels of sedation and prolonged bed immobilization to grant comfort and patient safety to critically ill children during their pediatric intensive care unit (PICU) stay (6–8).

A complementary bundle of practices known as the “ABCDEF” or “A-F” bundle, have been recommended as standard of care in adults by the Society of Critical Care Medicine (9, 10). This bundle -has been also referred to as ICU-based rehabilitation (11), as that rationale for this intervention is to prevent ICU-acquired morbidities, improve functional recovery and reduce p-PICS. Pediatric specific data are emerging (12, 13) on the feasibility and safety of PICU-based rehabilitation (12, 14, 15) and several PICUs are currently starting to implement the “A-F” bundle, adopting assessment screening tools for delirium, optimizing daily sedation and assessing patient readiness for mobilization (15–21). However, pragmatic data on how to implement this bundle, especially in limited resource PICU are scarce; further, since this bundle requires the adoption of several interventions, including environmental changes and investments, prioritization of clinical interventions is crucial for success where both human and physical resources are limited.

The main objective of this quality improvement project was to evaluate the feasibility of the implementation of a structured and interdisciplinary liberation protocol (LiberAction project), prioritizing three key domains: delirium screening and treatment, early mobilization and sedation in a human resource-limited PICU of a tertiary Italian children's hospital. As secondary objectives, we aimed to describe the type, timing and duration of delirium, the barriers encountered to implement early mobilization and the adverse events attributable to mobilization.

## Materials and Methods

### LiberAction Project Development

The objective of the LiberAction project was to implement a change in clinical care practices in a medical PICU (Area Rossa, Bambino Gesù, Children Hospital, Rome, Italy). This practice change consisted of: (a) the introduction of a delirium screening and treatment protocol, (b) the adoption of a new sedation protocol to limit the use of benzodiazepines, and (c) the implementation of an early (<72 h from PICU admission) and tiered mobilization protocol.

This project began in October 2019 with the creation of a core inter-professional working group consisting of a PICU physician, two nurses and a physiotherapist (LiberAction Working group). This core group was trained for 1 week at the PICU in McMaster Children's Hospital, Hamilton, Ontario, Canada, a site experienced in developing and implementing early rehabilitation specifically in critically ill children (6, 22). During the training period, this working group focused on obtaining hands-on experience on delirium prevention, screening and management, benzodiazepine-limited sedation strategies and early mobilization. This period was also focused in: (a) understanding how the institutional evidence-based practices were developed, (b) reviewing the educational resources, (c) observing how the bedside team communicated and applied these into practice and, (d) engaging with administrative leadership as well as with front line staff (nursing, allied health, and physician trainees) and patients' families, on the challenges and successes of PICU-based rehabilitation practices. At the end of this training period, a meeting with the McMaster PICU staff was instituted to evaluate how their rehabilitation program could have been applied to the PICU setting in Bambino Gesù, Italy.

Upon return to Italy, the LiberAction working group met weekly over the following 8 months (15th November 2019–30th June 2020) to develop a rehabilitation program tailored for a human resource-limited PICU. Three inter-professional subgroups were created, consisting of key stakeholders of pediatric critical care medicine, pediatric critical care nurses, and pediatric critical care rehabilitation therapists. Each subgroup was assigned a bundle (i.e., delirium screening/treatment, early mobilization and, sedation) and a team leader. The major challenges of each team were: (a) prepare specific guidelines on delirium screening and management, sedation and early mobilization (3, 23) tailored for the local needs, (b) educate physicians, nurses and physiotherapists with weekly meeting before the morning medical rounds during the pre-implementation period and, (c) discuss about the potential barriers and solving strategies to implement the LiberAction project. To achieve these goals, each subgroup conducted a comprehensive review of the published evidence in pediatrics.

Educational resources and on-line videos were developed and made available for all the PICU staff during the pre-implementation period. Finally, the core working group determined valid and feasible outcome measures, based on previously published pediatric data (12, 15, 24), to evaluate the LiberAction project during the implementation period (1st July 2020–31st December 2020).

The LiberAction project was implemented without any additional personnel or equipment, further, it was developed during the unplanned SARS-CoV-2 pandemic which spread in Italy at the end of February 2020.

### LiberAction Project Design

#### Setting

The LiberAction project was implemented in a 6-bed medical PICU of the Children's Hospital Bambino Gesù, a tertiary care pediatric hospital that cares children aged 18 years and under. The PICU is staffed by 28 full time staff consisting eight attending physicians, 16 registered nurses who provide care with a nurse-to-patient ratio of 1:2. Four physiotherapists and three psychologists who are not dedicated to the PICU, cross cover the unit, in addition to the other medical and surgical units. Both physiotherapists and psychologists consult and treat only when ordered by a PICU physician. As for internal policy, parents are allowed to stay with their child all day, except during the morning rounds due to space limitation.

During the pandemic, the PICU served as a “non-COVID-19 PICU” and was left opened to parents without symptoms suggestive of SARS-CoV-2 infection or not in obligatory home-isolation (25). From May 2020, parents required a negative rapid polymerase chain reaction test to have access to PICU. The test was performed directly in our hospital before entry.

#### Study Design

The LiberAction project was an observational pre-post implementation study, evaluated using a non-probability, convenience sampling technique (12). The project was reviewed and approved by our Medical Directorate as a quality improvement project and exempted from further review. Inclusion criteria were children aged between 1 day and 18 years who required PICU admission for more than 72 h. Patients were excluded if early mobilization was contraindicated [e.g., trauma patients with unstable fractures, brain injury at risk of intracranial hypertension or with elevated intracranial pressure (> 15 cm H_2_O during sedation), patients with refractory hypotension or respiratory failure requiring escalating therapies, patients receiving neuromuscular blocking agent, patients with uncontrolled bleeding or because of medical orders]. Patients admitted to the PICU from November 15th 2019 to June 30th 2020 served as control group in the pre-implementation period. Patients admitted in PICU from July 1st to December 31st 2020 served as intervention group in the implementation period.

The manuscript was prepared following Standards for Quality Improvement Reporting Excellence (SQUIRE) 2.0 guidelines (26).

#### The Intervention

The LiberAction project prioritized the implementation of the following three bundles of care: (a) a delirium screening and treatment protocol, (b) a benzodiazepine-limited sedation protocol, and (c) early mobilization (within the first 72 h of PICU admission) protocol.

#### Pre-intervention Practices (15th November 2019–30th June 2020)

**Figure 1:**
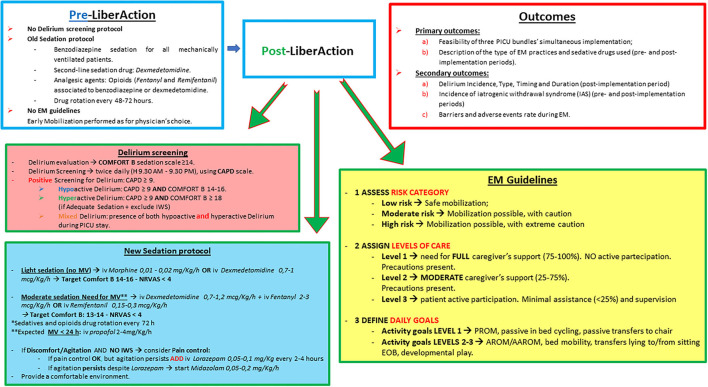
PICU practices in the pre/post-implementation period and study outcomes. EM, Early mobilization; IWS, Iatrogenic withdrawal syndrome; MV, mechanical ventilation; NRVAS, nurse reported visual analogic pain scale; PROM, passive range of motion; AROM, active range of motion; AAROM, assisted/active range of motion; iv, intravenous; PICU, pediatric intensive care unit.

Delirium was never screened in our PICU and its recognition was based only on clinical suspicion and psychiatric evaluation. The sedation protocol, driven by both physicians and nurses, consisted in the use of midazolam as first-line sedation drug in all children receiving mechanical ventilation (MV) for more than 24 h. Second-line sedation drug was dexmedetomidine. Opioids (morphine, fentanyl and remifentanil) were used for analgesia and were rotated every 48–72 h to avoid tachyphylaxis. A weaning protocol was used to reduce the risk of iatrogenic withdrawal syndrome (IWS) in patients receiving more than 5 days of sedation. IWS was assessed using the Withdrawal Assessment Tool version 1 (WAT-1) (3). Early mobilization was rarely performed and not protocolized (Figure 1).

#### Post-intervention Practices (1st July 2020–31st December 2020)

##### Delirium Assessment and Treatment

During the implementation period, delirium was screened twice daily (9.30 a.m. and 9.30 p.m. during the rounds; Figure 1) by the nurse in charge of the patient using the Cornell Assessment of Pediatric Delirium (CAPD) scale and its developmental anchor points for patients below 2 years (Supplementary Figure 1) (23). A CAPD score of 9 or higher represented a positive screen, however delirium positive screening was further confirmed by the physician in charge. In case of disagreement, consensus was obtained by discussion. Developmentally delayed child screened positive for delirium, prior to be classified as delirious, had to receive a confirmation by a psychiatrist. A child was considered developmentally delayed according to the past medical history provided by the parents or, if the Pediatric Cerebral Performance Category scale was 4 at PICU admission (24).

Patients were evaluated for delirium only when the sedation scale score of the COMFORT B (3, 27) was ≥14. Patients with a COMFORT B score < 14 were classified as unarousable to verbal stimuli (“pharmacologic coma”) and thus, not evaluated for delirium (Supplementary Figure 1).

Patients with a COMFORT B score ≥ 18 and CAPD > 9 were considered not adequately sedated and thus, re-evaluated for sedation, pain, or IWS before being considered as delirious (3).

Delirium subtypes were determined combining the level of sedation and the psychomotor activity. Positive screening for hypoactive delirium was considered when the CAPD score was ≥9 and the COMFORT B score between 14 and 16. Positive screening for hyperactive delirium was considered when the CAPD score was ≥9 and the COMFORT B score was ≥18 after having excluded pain, inadequate sedation and IWS (3). Positive screening for mixed delirium was considered when the patient, during 24 h of PICU stay, presented both hypoactive and hyperactive delirium.

Delirium treatment was performed using with non-pharmacologic interventions (Supplementary Figure 1). Pharmacologic interventions were used only when the first interventions failed (15, 24, 28).

##### Sedation and Analgesia

A benzodiazepine-sparing protocol and guidelines to wean patients sedated for more than 5 days were developed and adopted in the post-implementation period (Supplementary Figures 2, 3). Dexmedetomidine was introduced as first sedative agent in order to reduce the daily dose of benzodiazepines (21, 29). Sedation goals were arbitrarily set according to the COMFORT B scale for sedation and to the Nurse Reported Scale Visual Analogic Pain Scale (NRVAS) for pain (3, 27) during the morning round according to the patient's needs. Patients with a COMFORT B score between 13 and 17 were considered adequately sedated. Due to logistics (a low nurse-to-patient ratio of 1:2) and safety concerns (risk of extubation, loss of invasive devices and falls), our protocol did not include a daily sedation interruption (3).

In order to maximize patient comfort and further reduce pharmacologic interventions, several environmental PICU changes and clinical interventions were adopted during the post-implementation period: reduction of noises, liberation from physical restraints, sleep promotion and, family engagement in the PICU activities despite the SARS-CoV-2 pandemic hospital restrictions (18, 30). Median cumulative doses of sedatives (midazolam and dexmedetomidine) and opioids (fentanyl, remifentanil, and morphine) drugs corrected for the total number of sedation days in the pre- and post-implementation period were evaluated.

##### Early Mobilization

Early mobilization consisted of a range of graduated activities that were developmentally appropriate and individualized for each patient daily according to the clinical condition, strength and endurance. To provide a safe mobilization, we developed a tiered activity plan of mobilization according to three level of assistance (Supplementary Figures 4, 5). This tier plan ranged from a minimum of passive mobilization activities to prevent muscle wasting and optimize circulation, to a maximum of active mobilization activities to enhance muscle strength and prevent deconditioning. Criteria for interrupting, altering, or, aborting any form of mobilization therapy were also developed.

The mobilization plan (level 1–3) was daily discussed during the morning rounds. The approved plan was then recorded in the electronic chart and communicated to the physiotherapists. At the end of the day the nurse in charge of the patient reported in the electronic chart: (a) the number and the type of mobilizations per patient, (b) the duration of each session of mobilization, (c) if the parents actively participate to mobilization, (d) the barriers encountered to mobilization, (e) the adverse events attributable to mobilization (tube dislodgement, central catheter accidental removal, falls, etc.).

### Outcome Measures

The primary PICU outcomes of interest were as follows: (a) feasibility of the simultaneous implementation of the three bundles (PICU staff adherence); (b) description of the early mobilization practices and of the cumulative doses of analgo-sedative drugs used during the pre- and post-implementation periods. Secondary outcomes were the followings: (a) description of incidence, type, timing, and duration of delirium in the post-implementation period, (b) duration of MV, PICU length of stay and incidence of IWS during the pre- and post-implementation period; (c) description of the barriers encountered to mobilization and adverse events rate attributable to mobilization. Time to onset of delirium was defined as the number of days from PICU admission to the first positive screening for delirium. Duration of delirium was defined as the number of days in which the screening of delirium was positive.

At the end of the study (January 2021), PICU staff perceptions about the LiberAction project were evaluated using a self-administered survey.

### Statistical Analysis

Demographic and clinical data were reported as *n* (%) and median and interquartile range [IQR] for categorical and continuous variables, respectively. Comparisons before and after the implementation of the LiberAction project was performed using Fisher's exact test or Wilcoxon rank-sum test, as appropriate. A two-side *p* < 0.05 was considered as statistically significant. All the analysis was performed with GraphPad Prism 9 (San Diego, CA, USA).

## Results

Two hundred and twenty-five patients met the inclusion criteria. One hundred and thirty-seven in the pre-implementation period and 88 in the post-implementation period. SAR-CoV-2 pandemic did not limit the development of the LiberAction project, however, it caused an important reduction (50%) of PICU admissions compared with previous years. Patient characteristics in the pre- and post-implementation period are summarized in Table 1. Eighty children (58.4%) required MV in the pre-implementation period, while 53 (60.2%) in the post-implementation period.

**Table 1:** Patient characteristics.

	**Pre-implementation period** ***n* = 137**	**Post-implementation period** ***n* = 88**	***P***
	***(15th November 2019–30th June 2020)***	***1st July 2020–31st December 2020***	
Age in years	4.20 (2.20–6.00)	3.30 (2.20–5.20)	0.04
Weight in Kg	16.5 (12.30–18.50)	15.50 (12.30–17.50)	0.09
**Gender** ***n*** **(%)**			
Male	65 (47.4)	40 (45.5)	0.78
Female	72 (52.6)	48 (54.6)	
Admitting diagnosis category	Respiratory failure: 60 (43.8)	Respiratory failure: 32 (36.4)	0.06
	Septic shock: 42 (30.7)	Septic shock: 26 (29.6)	
	Renal/metabolic disorders: 20 (14.6)	Renal/metabolic disorders: 18 (20.5)	
	Cardiogenic shock: 5 (3.7)	Cardiogenic shock: 10 (11.4)	
	Trauma: 10 (7.3)	Trauma: 2 (2.3)	
Patients with developmental delay *n* (%)	20 (14.6)	12 (13.6)	0.99
PIM 2 score	3.35 (2.18–26.10)	3.70 (2.19–25.00)	0.72
Patients requiring MV *n* (%)	80 (58.4)	53 (60.2)	0.88
Patients requiring vasoactive medications *n* (%)	56 (40.9)	33 (37.1)	0.67
Patients requiring neuromuscular blocking agents *n* (%)	18 (13.1)	10 (11.4)	0.84
MV duration (days)	5.20 (5.30–7.00)	4.30 (1.50–6.20)	0.0001
PICU LOS (days)	7.50 (5.00–9.00)	6.20 (4.50–8.50)	0.0003
PICU mortality *n* (%)	7 (5.1)	6 (6.8)	0.57

*Continuous data are reported as median and interquartile range [IQR], categorical variables are reported as n (%)*.

*PICU, pediatric intensive care unit; PIM2, pediatric index of mortality 2; MV, mechanical ventilation; LOS, length of stay*.

### Primary Outcomes

#### Sedation Practice

Adherence to the new sedation protocol was reported in 43 of the 53 sedated children (81.1%). Ten (18.9%) children received continuous benzodiazepine infusion for >48 h since the beginning of MV as for physician's in charge choice.

Median cumulative dose (mg/kg/sedative days) of benzodiazepines (midazolam and lorazepam) were significantly lower in the post-implementation period compared with the pre-implementation period: [0.83 (IQR: 0.53–1.31) vs. 0.74 (IQR: 0.55–1.16), *p* = 0.0001] (Table 2), while the opposite was for dexmedetomidine (mcg/kg/sedative days): [0.53 (IQR: 0.40–0.70) vs. 2.64 (IQR: 1.96–3.47), *p* = 0.0001]. Median cumulative doses of fentanyl, remifentanil and morphine (mcg/kg/sedative days) were lower in the post-implementation period, but these differences were not significant.

**Table 2:** Descriptive analysis of sedation/analgesia and early mobilization practices between the pre-post implementation period.

	**Pre-implementation period** ***n* = 137**	**Post-implementation period** ***n* = 88**	***P***
	***(15th November 2019–30th June 2020)***	***1st July 2020–31st December 2020***	
**Sedation/analgesic drugs**			
Benzodiazepine^*^ (mg/kg/sedation days)	0.83 (0.53–1.31)	0.74 (0.55–1.16)	0.0001
Dexmedetomidine^*^ (mcg/kg/sedation days)	0.53 (0.40–0.70)	2.64 (1.96–3.47)	0.0001
Fentanyl^*^ (mcg/kg/sedation days)	26.34 (17.49–34.68)	20.16 (17.39–27.36)	0.07
Remifentanil^*^ (mcg/kg/sedation days)	83.70 (56.92–119.52)	67.50 (49.50–102.15)	0.24
Morphine^*^ (mcg/kg/sedation days)	14.61 (10.16–22.69)	12.67 (9.50–15.84)	0.06
Patients with iatrogenic withdrawal syndrome (WAT-1>3) *n* (%)	33 (41.3)	11 (20.8)	0.015
**Early mobilization**			
Adherence *n* (%)	35 (25.6)	62 (70.5)	0.0001
Number of mobilizations per patient in the first 72 h	3.00 (2.00–4.00)	7.00 (3.00–12.00)	0.004
Duration of mobilization per patient (minutes)	4.00 (3.50–4.50)	5.50 (5.25–6.50)	0.0001
Number of passive mobilizations per patient in the first 72 h	3.00 (2.00–3.75)	10.00 (9.00–12.00)	0.0001
Number of active mobilizations per patient in the first 72 h	1.50 (1.00–2.00)	3.00 (2.00–3.00)	0.0007
Number of patients mobilized out of bed *n* (%)	8 (5.8)	10 (11.4)	0.20
Number of patients mobilized with the support of the family *n* (%)	10 (7.3)	33 (37.5)	0.0001

*Continuous data are reported as median and interquartile range [IQR], categorical variables are reported as n (%)*.

* *Median cumulative dose of sedative/analgesic drugs corrected for the total number of sedation days used during PICU stay; WAT-1, Withdrawal Assessment Toll version 1*.

#### Early Mobilization

Sixty-two (70.5%) children received early mobilization in the post-implementation period compared with thirty-five (25.6%) in the pre-implementation period (Table 2).

The median number per patient and the duration of each mobilization significantly increased between the two study periods [3.00 (IQR: 2.00–4.00) vs. 7.00 (IQR: 3.00–12.00); *p* = 0.004 and 4 min (IQR: 3.50–4.50) vs. 5.50 min (IQR: 5.25–6.50); *p* < 0.0001, respectively]. In-bed passive mobilizations represented the majority of the mobilizations in both periods, however, both passive and active mobilization significantly increased when comparing the two study periods (Table 2). Ambulation (with or without intubation) was not feasible since the limited spaces of the unit. Family engagement in early mobilization practices significantly increased in the post-implementation period compared with the pre-implementation period (7.3 vs. 37.5%; *p* < 0.0001).

#### Delirium Screening and Treatment

Adherence to delirium screening was 90.9% (80 of 88 patients received at least one delirium evaluation every day during the PICU stay).

### Secondary Outcomes

Twenty children (23%) screened positive for delirium; all of whom were invasively MV. Nine (45%) children were aged <2 years and 7 (35%) were developmentally delayed. Time to onset of delirium was on the third day after PICU admission (IQR: 2.00–4.00) and lasted 2 days (IQR: 1.00–3.00). When assessed by phenotype, 15 (75%) positive screened children reported a hypoactive delirium, 3 (15%) a hyperactive delirium and 2 (10%) a mixed delirium. None reported recurrent episodes of delirium. In the 20 children screened positive for delirium, non-pharmacologic interventions for delirium were applied, only two (10%) required pharmacologic treatments with antipsychotics (one patient with hypoactive delirium received risperidone and the other one with hyperactive delirium received haloperidol).

Median duration of MV (days) and of PICU stay (days) were significantly lower in the post-implementation period compared with the pre-implementation period (Table 1). Occurrence of IWS was significantly higher in the pre-implementation period compared with the post-implementation period (Table 2).

Barriers to early mobilization were: disease severity and bed rest orders (55%), lack of physicians' order (20%), lack of human resources (20%), and lack of adequate devices for patient mobilization (5%). Adverse events related to early mobilization were not reported in both periods, however, early mobilization was aborted in 6 (11.32%) children due to a sudden hemodynamic/respiratory impairment in the post-implementation period.

### PICU Staff Perceptions of the LiberAction Project

**Figure 2:**
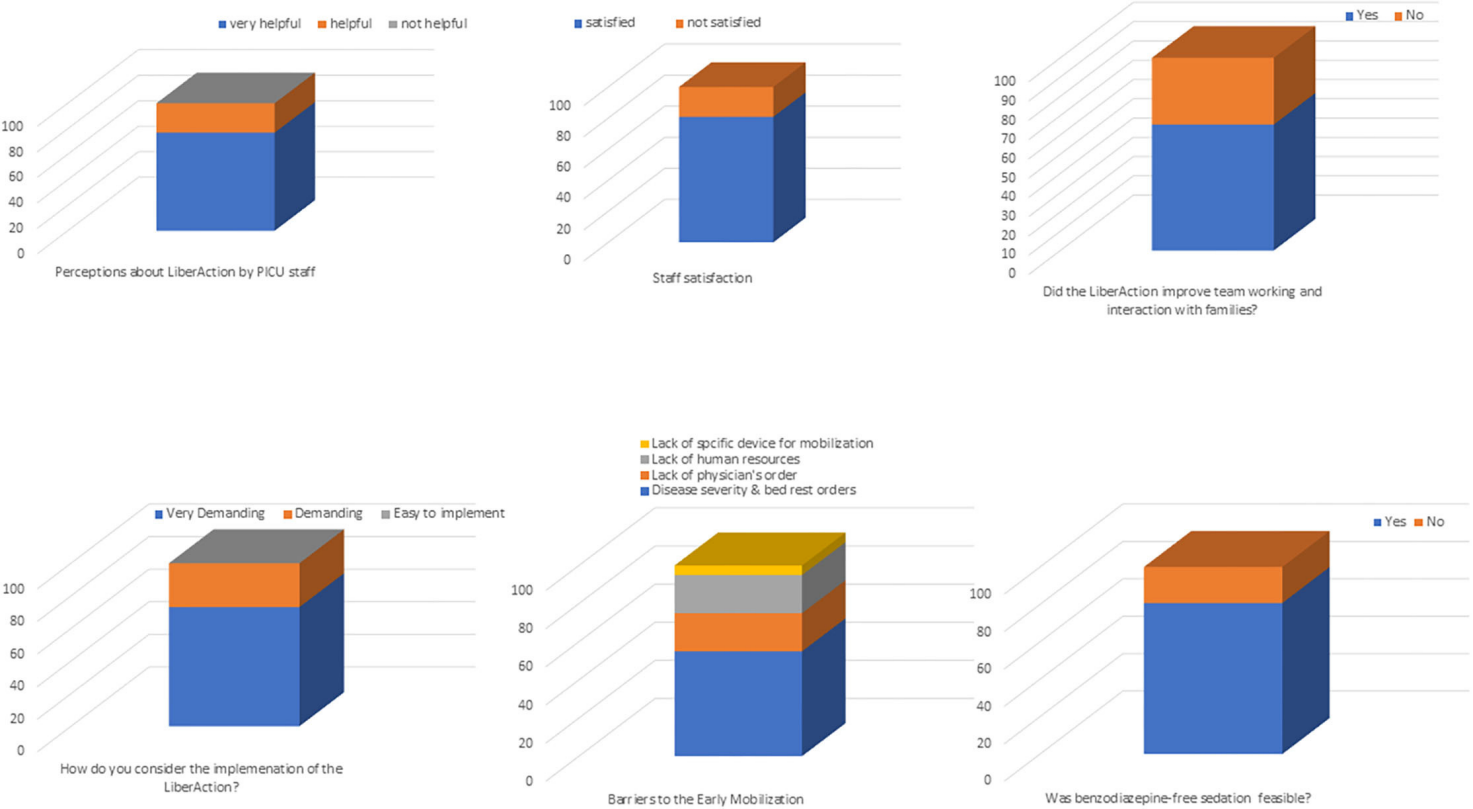
PICU staff perceptions of the LiberAction project.

Twenty-eight questionnaires were sent to all PICU staff. Of these, 26 (92.9 %) were completed and used for the evaluation. Main results are reported in Figure 2.

Twenty (76.9%) respondents evaluated the LiberAction project as a “very helpful initiative,” the remaining six respondents (23.1%) as “helpful” to improve patients' outcomes. Twenty-one (80.8%) were satisfied with bundle implementation and 23 (88.5%) agreed that the three bundle components should be considered standard of care in the PICU. Seventeen (65.4%) perceived that the introduction of the bundle in PICU improved inter-professional team collaboration, interactions with families and families' satisfaction.

Key facilitators included the followings: 17 (65.4%) respondents considered physiotherapists as an essential figure “to start” an early mobilization program; 22 (84.6%) respondents considered the nurse staff as an essential figure “to sustain” a mobilization program. Family engagement during mobilization was considered helpful by 20 (76.9%) respondents, while six (23.1%) remained uncertain. All respondents (100%) agreed that the number of mobilizations increased since the introduction of a dedicated mobilization protocol. All respondents (100%) considered as “very useful” the screening of delirium and the introduction of a new sedation protocol, however, twenty (80.8%) retained that the use of benzodiazepine sedation was still essential to manage more complex patients. Thirteen (50%) respondents reported that delirium screening was more difficult in patients below 2 years old and developmentally delayed.

Perceived barriers were as follows: 19 (73.1%) respondents considered the LiberAction project as “very demanding” in terms of requiring human and physical resources. Lack of resources was the commonest barrier to mobilization (100% respondents). All respondents (100%) retained that our PICU was logistically inadequate to develop the LiberAction project (e.g., limited spaces for mobilization, inadequate spaces for parents stay inside the PICU). Further, eight (30.8%) respondents reported reluctancy to mobilize patients viewing mobilization not as a priority of the therapeutic plan.

## Discussion

To the best of our knowledge, this is the first study to report on the “contemporary” implementation of the three major components of the ICU Liberation Bundle (delirium screening and treatment, early mobilization and sedation management) in a human resource-limited PICU. Clinical implementation of these three practices was feasible and safe regardless barriers related to the shortage of human and physical resources and SARS-CoV-2 restrictions. One week of on-site training at the McMaster PICU, was helpful to develop protocols tailored to our local setting and resources, speed up the educational process (8 months) among PICU staff, reduce benzodiazepine use for sedation and, increase the early mobilization practices and family engagement.

In the last decade, advances in pediatric critical care significantly decreased mortality rates, however, this paralleled with an increase of children surviving with important functional morbidities (13, 28). Delirium is a common complication of pediatric critical illness and favors the development of p-PICS (5), thus, strategies to reduce delirium risk factors, such as prolonged immobilization and benzodiazepine use (10, 28, 31, 32), are now recommended by the Society of Critical Care Medicine ICU Liberation Campaign (10) to ensure optimal functional recovery in children after PICU stay.

In our unit, before the implementation of the LiberAction, delirium screening was never performed, early mobilization was not protocolized and sedation was mainly based on benzodiazepine, therefore, an extensive educational process of all PICU staff was needed to address important knowledge gaps (15). Compared with other similar quality improvement projects (12, 15, 21, 33), our educational process was relatively short (8 months), however, this time was sufficient to implement simultaneously all the three components of the PICU liberation bundle and achieve good results in terms of bundle adherence and patients' safety. These good achievements were possible mainly for two reasons: (a) the “*in situ* training” performed at the McMaster PICU in 2019 and (b) the limited number of PICU staff to be educated and trained. With the “*in situ* training,” the LiberAction core group learned: (a) how to *proportionately* apply the PICU bundle into our clinical practice [e.g., use of the COMFORT B scale instead of the RASS scale (34), focus on in-bed mobilization practices instead of ambulation because of our limited spaces, etc.] (b) which component of the PICU bundle prioritize and, (c) how to speed up PICU staff education. This educational process was also facilitated by the limited number of our PICU staff. Organizational and cultural changes, in fact, are more difficult to be accepted in large PICU units (35). Thus, the inconvenience of working in a human resource-limited PICU became a practical advantage in terms of education and training.

In the post-implementation period, adherence to delirium screening was high (90.9%) and this was consistent with previous findings of Simone et al. (15) and Valdivia et al. (36) showing that delirium screening could be easily and reliably performed with CAPD by PICU nurses. Delirium incidence in our unit was 23%, with hypoactive delirium as the most common phenotype (75%). Delirium incidence was similar with the one reported by other medical PICUs (15, 28, 36). In the majority of the cases, delirium was treated with non-pharmacologic interventions. Antipsychotics, which are off-label in pediatrics, were rarely used in our cohort (10%). These drugs were only used when non-pharmacologic interventions failed and when their chances of success where high. Notably, the lower incidence of the hyperactive phenotype in our cohort may be probably explained by considering our sedation protocol, which includes both sedative and opioid drugs for all children receiving MV > 24 h. This sedation practice was arbitrarily chosen to safely work with a nurse-to-patient ratio of 1:2.

Benzodiazepines have been the mainstay of pediatric sedation for many decades, however, in recent years a causal and temporal relationship was demonstrated between benzodiazepine exposure and delirium development (21, 29). The LiberAction project successfully implemented a benzodiazepine sparing sedation protocol. This was reflected in the decreased benzodiazepine use, and an accompanying increased use of dexmedetomidine post. Adherence to these sedation guidelines was high (81.2%). Deviations from this practice were reported in 18.9% of the sedated patients in the post-implementation period. Deviations were likely due to preference of benzodiazepine use in more complex cases or in children considered “a priori” difficult to sedate (e.g., developmentally delayed children or with a medical history of epilepsy where benzodiazepine are preferred more for therapeutic purposes than for sedation). In the post-implementation period there was a significant reduction of not only benzodiazepine use, but also opiates compared with pre-implementation period (3). This is likely the explanation for the reduced incidence of IWS, MV duration and PICU length of stay observed in the post-implementation period, however, we caution the interpretation of these results as this study design does not allow us to determine if this is directly related to the reduced sedation use.

The LiberAction project resulted in significant improvements in early mobilization in the post-implementation period. Mobilization was rarely performed before the implementation of the LiberAction project (25.6%) and consisted in the consultations of multiple rehabilitation services (physiotherapists dedicated to physical or respiratory therapy) without a dedicated protocol. The raised awareness of the risks associated with immobility and the introduction of a safe mobilization protocol increased early mobilization practices in our PICU with an adherence of 70.5%. Consistent with Wieczoreck et al. (12), the median number of mobilizations (both passive and active) per patient significantly increased during the post-implementation period without adverse events. Unfortunately, ambulation was unfeasible in our PICU due to the limited spaces, the lack of an adequate number of dedicated physiotherapists and, a nurse-to-patient ratio of 1:2. However, despite these barriers, in-bed activities (transfer from lying to sitting position, in-bed cycling, use of Wii, etc.) significantly increased in the post-implementation period. Of note, due to a proper SARS-CoV-2 surveillance (30, 37), family's access to our PICU was granted and this favored family engagement in all mobilization activities, especially when the nurses and physiotherapists' work load was high and some sessions risked to be missed. Based on these findings, we speculate that allowing parents to stay in PICU during SARS-CoV-2 pandemic kept the incidence of delirium in line with previous reports (16). An adult study performed in this period showed that pandemic restrictions may have increased delirium incidence in ICU (38).

The staff perceptions regarding the LiberAction project were overall positive. Consistent with other studies (12, 13), all the respondents to our questionnaire recognized the clinical importance of all the three components of the LiberAction project. However, this cultural change was challenging and very demanding for the majority of respondents. Lack of human and physical resources may in part explain this finding. In our country, respiratory therapists managing MV, occupational therapists and social workers are not present, and the majority of the rehabilitation workload is upon the physiotherapists and the nursing staff (12). Despite these barriers, several factors may have contributed to the implementation success: (a) the awareness of creating a healing environment without using drugs, (b) the inter-professional collaboration among physicians, nurses, physiotherapists and families and, (c) the improvement of the therapeutic alliance between the PICU staff and the family (39). All these aspects helped to overcome the barriers related to the lack of human and physical resources and created a momentum for change.

This study presents several important limitations. First, it is a single-center study where data are collected from a small convenience sample size. Thus, further research is needed to establish the generalizability of our findings. Second, our study was not intended to evaluate any association between the implementation of the ICU bundle and outcomes (reduction of IWS, duration of MV, PICU length of stay and mortality). There is an intricate relationship among benzodiazepine use, opiate use, MV and delirium which requires a different study design to be elucidated. Thus, it is possible that the reduction of duration of MV and of PICU stay observed in the post-implementation period could be due to other factors that were not captured by the patient characteristics and PIM 2 score. Third, we included in the study only children who stayed in PICU for more than 3 days, this limited the generalizability of our results for children requiring short PICU stay. Fourth, this study did not evaluate the workload (human resources used, extra-time dedicated to this project, etc.) and related costs requested to implement the LiberAction, the strategies used to overcome the lack of resources, the perceived barriers and the impact of this project on the post-intensive care syndrome. These aspects are relevant considering that hospital administrators require efficacy data before increasing resources (19, 40).

In conclusion, this study showed that the implementation of a multicomponent and interdisciplinary quality improvement project including delirium screening and treatment, limited-benzodiazepine sedation and early mobilization was feasible and safe in a resource-limited PICU and in time of pandemic. Further pediatric studies are needed to identify interventions designed to decrease delirium rates, evaluate the impact of both benzodiazepine-free sedation and early mobilization protocols on long-term functional outcomes.

## Data Availability Statement

The raw data supporting the conclusions of this article will be made available by the authors, without undue reservation.

## Ethics Statement

This Quality Improvement Project was approved by our Medical Directorate. Ethical review and approval was not required for the study on human participants in accordance with the local legislation and institutional requirements. Written informed consent to participate in this study was provided by the participants' legal guardian/next of kin.

## Author Contributions

MD, OG, and KC contributed to the conception and design of the work. MD and KC analyzed and interpreted the data. FBo, FBr, FS, FC, TS, LG, GZ, SP, GB, and MF contributed to data acquisition and the analysis and interpretation of the results. AB, IP, VR, and PL contributed to the data acquisition related to the early mobilization program. FBo, AR, and PT contributed to the data acquisition and analysis of the internal survey. DB contributed to the statistical analysis. AV, MR, and CC revised the work for important intellectual content. All authors revised the final article and approved the submitted version.

## Conflict of Interest

The authors declare that the research was conducted in the absence of any commercial or financial relationships that could be construed as a potential conflict of interest.

## Publisher's Note

All claims expressed in this article are solely those of the authors and do not necessarily represent those of their affiliated organizations, or those of the publisher, the editors and the reviewers. Any product that may be evaluated in this article, or claim that may be made by its manufacturer, is not guaranteed or endorsed by the publisher.

## References

[1] Pinto NP Rhinesmith EW Kim TY Ladner PH Pollack MM. Long-Term function after pediatric critical illness: results from the survivor outcomes study. Pediatr Crit Care Med. (2017) 18:e122–30. 10.1097/PCC.0000000000001070 28107265

[2] Walker TC Kudchadkar SR. Early mobilization in the pediatric intensive care unit. Transl Pediatr. (2018) 7:308–13. 10.21037/tp.2018.09.02 30460183 PMC6212381

[3] Harris J Ramelet AS van Dijk M Pokorna P Wielenga J Tume L. Clinical recommendations for pain, sedation, withdrawal and delirium assessment in critically ill infants and children: an ESPNIC position statement for healthcare professionals. Intensive Care Med. (2016) 42:972–86. 10.1007/s00134-016-4344-1 27084344 PMC4846705

[4] Herrup EA Wieczorek B Kudchadkar SR. Characteristics of postintensive care syndrome in survivors of pediatric critical illness: a systematic review. World J Crit Care Med. (2017) 6:124–34. 10.5492/wjccm.v6.i2.124 28529914 PMC5415852

[5] Manning JC Pinto NP Rennick JE Colville G Curley MAQ. Conceptualizing post intensive care syndrome in children-The PICS-p framework. Pediatr Crit Care Med. (2018) 19:298–300. 10.1097/PCC.0000000000001476 29406379

[6] Choong K Canci F Clark H Hopkins RO Kudchadkar SR Lati J. Practice recommendations for early mobilization in critically ill children. J Pediatr Intensive Care. (2018) 7:14–26. 10.1055/s-0037-1601424 31073462 PMC6260323

[7] Kudchadkar SR Yaster M Punjabi NM. Sedation, sleep promotion, and delirium screening practices in the care of mechanically ventilated children: a wake-up call for the pediatric critical care community^*^. Crit Care Med. (2014) 42:1592–600. 10.1097/CCM.0000000000000326 24717461 PMC4061156

[8] Ista E Scholefield BR Manning JC Harth I Gawronski O Bartkowska-Sniatkowska A. Mobilization practices in critically ill children: a European point prevalence study. (EU PARK-PICU). Crit Care. (2020) 24:368. 10.1186/s13054-020-02988-2 32576273 PMC7311184

[9] Ely EW. The ABCDEF bundle: science and philosophy of how ICU liberation serves patients and families. Crit Care Med. (2017) 45:321–30. 10.1097/CCM.0000000000002175 28098628 PMC5830123

[10] Pun BT Balas MC Barnes-Daly MA Thompson JL Aldrich JM Barr J. Caring for critically ill patients with the ABCDEF bundle: results of the ICU liberation collaborative in over 15,000 adults. Crit Care Med. (2019) 47:3–14. 10.1097/CCM.0000000000003482 30339549 PMC6298815

[11] Choong K. PICU-acquired complications: the new marker of the quality of care. ICU Manage Pract. (2019) 19:85–8. Available online at: https://healthmanagement.org/c/icu/issuearticle/picu-acquired-complications-the-new-marker-of-the-quality-of-care31763206

[12] Wieczorek B Ascenzi J Kim Y Lenker H Potter C Shata NJ. PICU up!: impact of a quality improvement intervention to promote early mobilization in critically ill children. Pediatr Crit Care Med. (2016) 17:e559–66. 10.1097/PCC.0000000000000983 27759596 PMC5138131

[13] Patel RV Redivo J Nelliot A Eakin MN Wieczorek B Quinn J. Early mobilization in a PICU: a qualitative sustainability analysis of PICU up! Pediatr Crit Care Med. (2021) 22:e233–42. 10.1097/PCC.0000000000002619 33315754 PMC8016701

[14] Walker T Kudchadkar SR. Early mobility in the pediatric intensive care unit: can we move on? J Pediatr. (2018) 203:10–2. 10.1016/j.jpeds.2018.08.058 30270161

[15] Simone S Edwards S Lardieri A Walker LK Graciano AL Kishk OA. Implementation of an ICU bundle: an interprofessional quality improvement project to enhance delirium management and monitor delirium prevalence in a single PICU. Pediatr Crit Care Med. (2017) 18:531–40. 10.1097/PCC.0000000000001127 28410275

[16] Traube C Silver G Reeder RW Doyle H Hegel E Wolfe HA. Delirium in critically ill children: an international point prevalence study. Crit Care Med. (2017) 45:584–90. 10.1097/CCM.0000000000002250 28079605 PMC5350030

[17] Meyburg J Dill ML von Haken R Picardi S Westhoff JH Silver G. Risk factors for the development of postoperative delirium in pediatric intensive care patients. Pediatr Crit Care Med. (2018) 19:e514–21. 10.1097/PCC.0000000000001681 30059477

[18] Kawai Y Weatherhead JR Traube C Owens TA Shaw BE Fraser EJ. Quality improvement initiative to reduce pediatric intensive care unit noise pollution with the use of a pediatric delirium bundle. J Intensive Care Med. (2019) 34:383–90. 10.1177/0885066617728030 28859578

[19] Treble-Barna A Beers SR Houtrow AJ Ortiz-Aguayo R Valenta C Stanger M. PICU-based rehabilitation and outcomes assessment: a survey of pediatric critical care physicians. Pediatr Crit Care Med. (2019) 20:e274–82. 10.1097/PCC.0000000000001940 30946294 PMC7132781

[20] Silver G Doyle H Hegel E Kaur S Mauer EA Gerber LM. Association between pediatric delirium and quality of life after discharge. Crit Care Med. (2020) 48:1829–34. 10.1097/CCM.0000000000004661 33031144 PMC8195312

[21] Shildt N Traube C Dealmeida M Dave I Gillespie S Moore W. “Difficult to Sedate”: successful implementation of a Benzodiazepine-Sparing Analgosedation-Protocol in mechanically ventilated children. Children. (2021) 8:348. 10.3390/children8050348 33924822 PMC8146538

[22] PICU Liber 8. PICU LIBER8 Bundle (2021). Available online at: https://piculiber8.com/piculiber8-in-action (accessed May 2021).

[23] Traube C Silver G Kearney J Patel A Atkinson TM Yoon MJ. Cornell assessment of pediatric delirium: a valid, rapid, observational tool for screening delirium in the PICU^*^. Crit Care Med. (2014) 42:656–63. 10.1097/CCM.0b013e3182a66b76 24145848 PMC5527829

[24] Traube C Silver G Gerber LM Kaur S Mauer EA Kerson A. Delirium and mortality in critically ill children: epidemiology and outcomes of pediatric delirium. Crit Care Med. (2017) 45:891–8. 10.1097/CCM.0000000000002324 28288026 PMC5392157

[25] Alhazzani W Evans L Alshamsi F Møller MH Ostermann M Prescott HC. Surviving sepsis campaign guidelines on the management of adults with Coronavirus Disease 2019 (COVID-19) in the ICU: first update. Crit Care Med. (2021) 49:e219–e34. 10.1097/CCM.0000000000004899 33555780

[26] Ogrinc G Davies L Goodman D Batalden P Davidoff F Stevens D. Squire 2.0 (Standards for quality improvement reporting excellence): revised publication guidelines from a detailed consensus process. Am J Crit Care. (2015) 24:466–73. 10.4037/ajcc2015455 26523003

[27] Boerlage AA Ista E Duivenvoorden HJ de Wildt SN Tibboel D van Dijk M. The COMFORT behaviour scale detects clinically meaningful effects of analgesic and sedative treatment. Eur J Pain. (2015) 19:473–9. 10.1002/ejp.569 25070754

[28] Siegel EJ Traube C. Pediatric delirium: epidemiology and outcomes. Curr Opin Pediatr. (2020) 32:743–9. 10.1097/MOP.0000000000000960 33105274

[29] Kamat PP Kudchadkar SR. IV clonidine in the PICU: time for dexmedetomidine to share the limelight? Pediatr Crit Care Med. (2018) 19:792–4. 10.1097/PCC.0000000000001649 30095722

[30] Mistraletti G Giannini A Gristina G Malacarne P Mazzon D Cerutti E. Why and how to open intensive care units to family visits during the pandemic. Crit Care. (2021) 25:191. 10.1186/s13054-021-03608-3 34078445 PMC8171999

[31] Mody K Kaur S Mauer EA Gerber LM Greenwald BM Silver G. Benzodiazepines and development of delirium in critically ill children: estimating the causal effect. Crit Care Med. (2018) 46:1486–91. 10.1097/CCM.0000000000003194 29727363 PMC6095819

[32] Silver G Traube C Gerber LM Sun X Kearney J Patel A. Pediatric delirium and associated risk factors: a single-center prospective observational study. Pediatr Crit Care Med. (2015) 16:303–9. 10.1097/PCC.0000000000000356 25647240 PMC5031497

[33] Dervan LA Di Gennaro JL Farris RWD Watson RS. Delirium in a tertiary PICU: risk factors and outcomes. Pediatr Crit Care Med. (2020) 21:21–32. 10.1097/PCC.0000000000002126 31568239

[34] Sessler CN Gosnell MS Grap MJ Brophy GM O'Neal PV Keane KA. The richmond agitation-sedation scale: validity and reliability in adult intensive care unit patients. Am J Respir Crit Care Med. (2002) 166:1338–44. 10.1164/rccm.2107138 12421743

[35] Porter-O'Grady T Malloch K. The emerging principles and practices of appreciative leadership. Nurs Manage. (2021) 52:16–22. 10.1097/01.NUMA.0000771732.53948.85 34469376

[36] Valdivia HR Carlin KE. Determining interrater reliability of the cornell assessment of pediatric delirium screening tool among PICU nurses. Pediatr Crit Care Med. (2019) 20:e216–e20. 10.1097/PCC.0000000000001896 30730379

[37] Liu K Nakamura K Katsukawa H Elhadi M Nydahl P Ely EW. ABCDEF bundle and supportive ICU practices for patients with Coronavirus Disease 2019 infection: an international point prevalence study. Crit Care Explor. (2021) 3:e0353. 10.1097/CCE.0000000000000353 33786432 PMC7994035

[38] Pun BT Badenes R Heras La Calle G Orun OM Chen W Raman R. Prevalence and risk factors for delirium in critically ill patients with COVID-19 (COVID-D): a multicentre cohort study. Lancet Respir Med. (2021) 9:239–50. 10.1016/S2213-2600(20)30552-X 33428871 PMC7832119

[39] Kleinpell R Zimmerman J Vermoch KL Harmon LA Vondracek H Hamilton R. Promoting family engagement in the ICU: experience from a national collaborative of 63 ICUs. Crit Care Med. (2019) 47:1692–8. 10.1097/CCM.0000000000004009 31567354

[40] Zheng K Sarti A Boles S Cameron S Carlisi R Clark H. Impressions of early mobilization of critically ill children-clinician, patient, and family perspectives. Pediatr Crit Care Med. (2018) 19:e350–e7. 10.1097/PCC.0000000000001547 29649021

