# Supplementary material for: The LiberAction Project: Implementation of a Pediatric Liberation Bundle to Screen Delirium, Reduce Benzodiazepine Sedation, and Provide Early Mobilization in a Human Resource-Limited Pediatric Intensive Care Unit
Source: Front Pediatr. 2021 Dec 8;9:788997. doi: 10.3389/fped.2021.788997 (PMC8692861; doi:10.3389/fped.2021.788997)
Supplement: Supplementary file 1 [file Data_Sheet_1.DOCX]

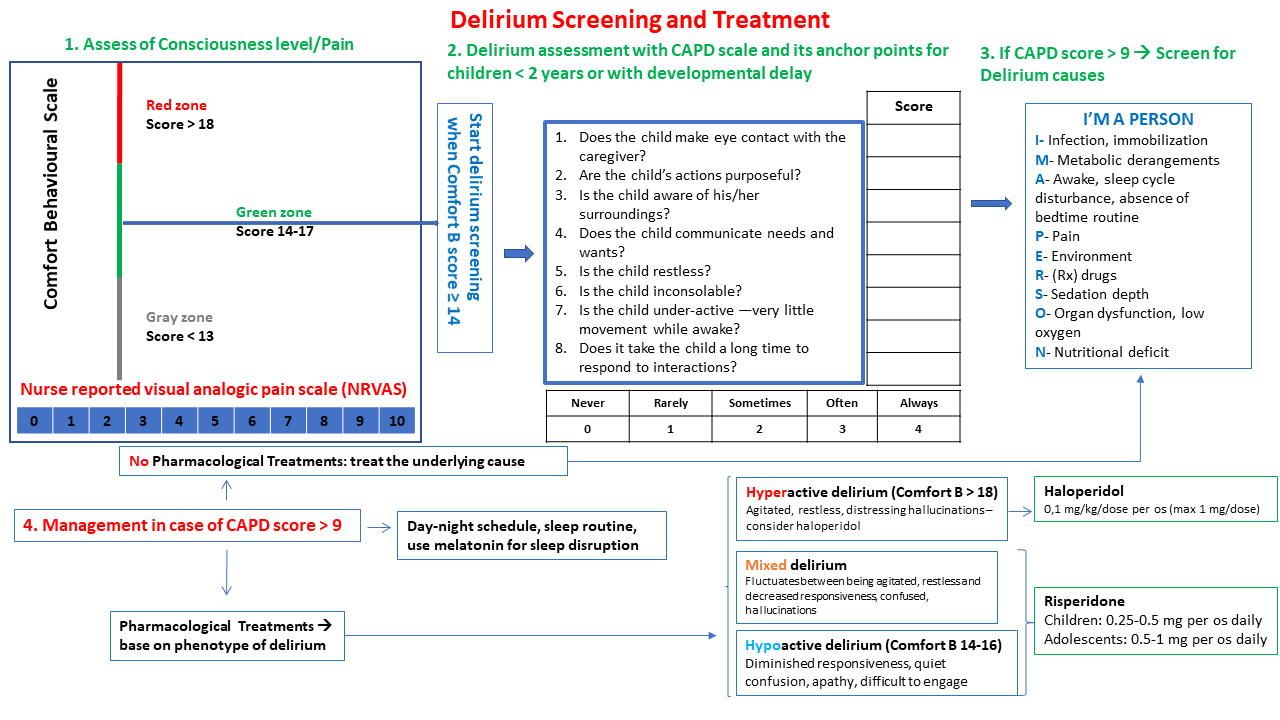


Figure 1 Supplemental material: Screening and Management of Delirium in the PICU


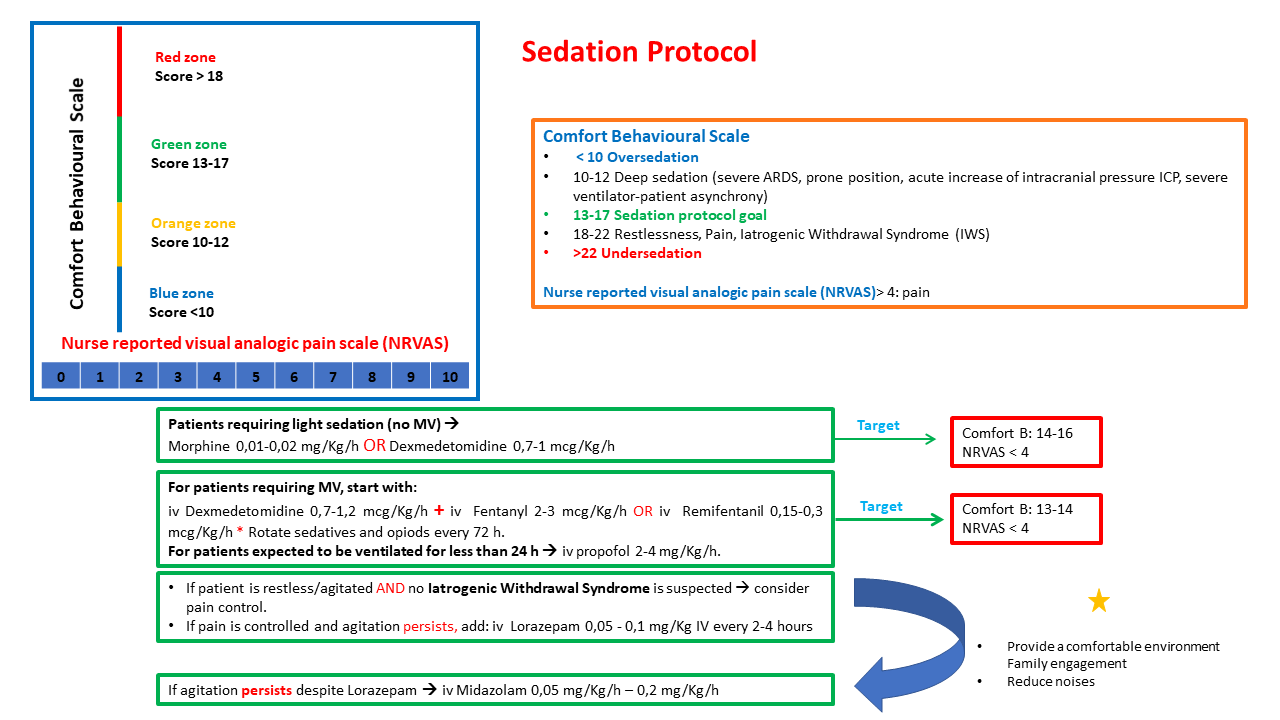


Figure 2 Supplemental material: Sedation protocol during the post-implementation period

Iv: intravenous; MV: Mechanical Ventilation; NRVAS: nurse reported visual analogic pain scale


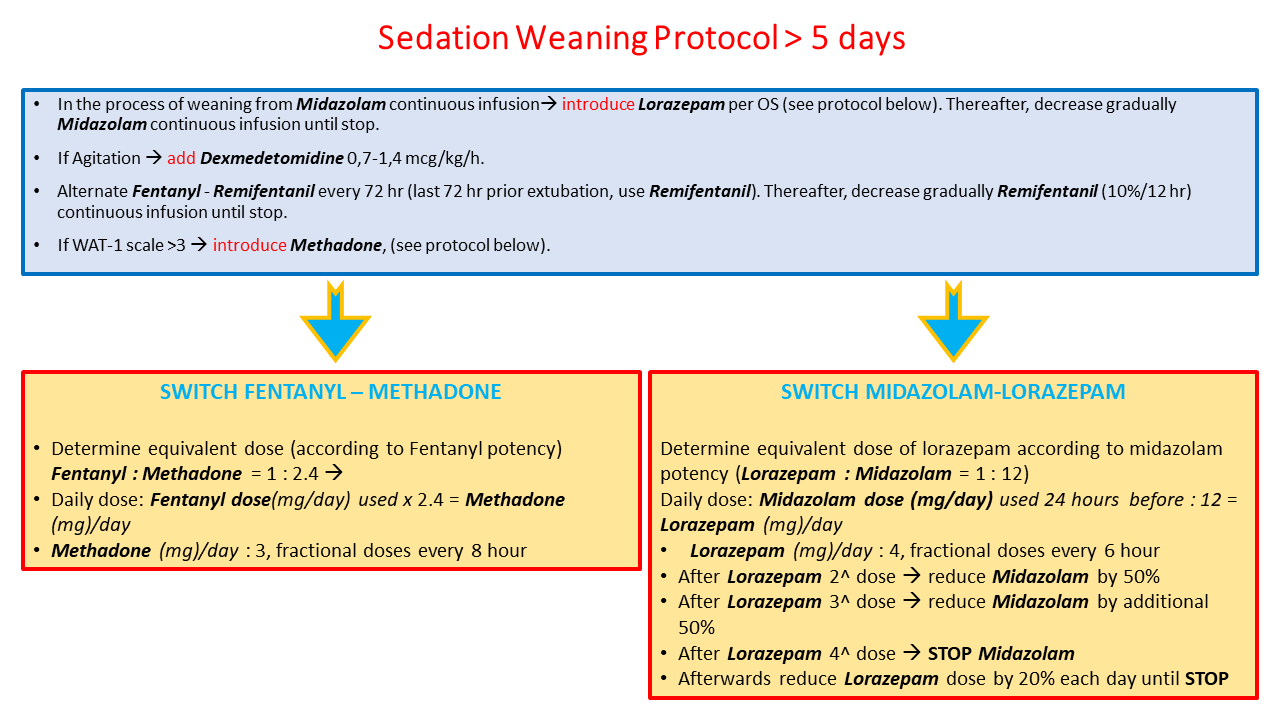


Figure 3 Supplemental material: Weaning protocol during the post-implementation period. WAT-1: Withdrawal assessment tool


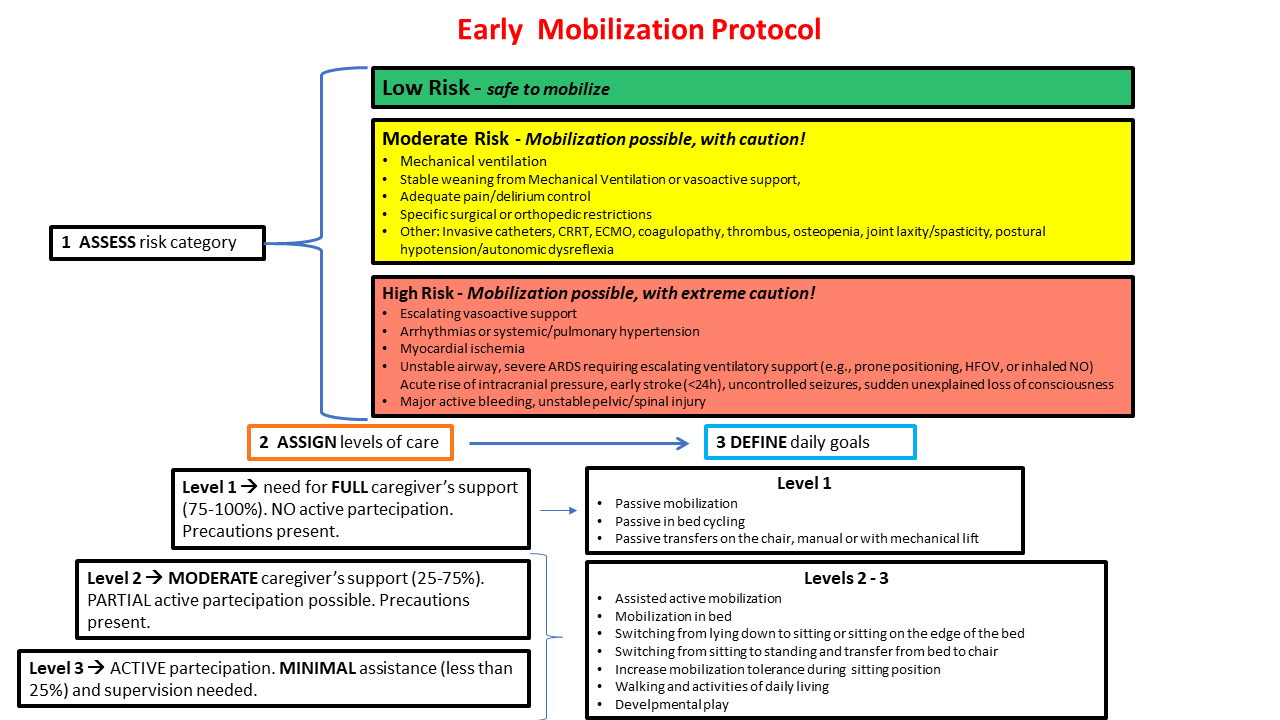


Figure 4 Supplemental material: Mobilization plan and tiered activity

CRRT: continuous renal replacement therapy; ECMO: extracorporeal membrane oxygenation;


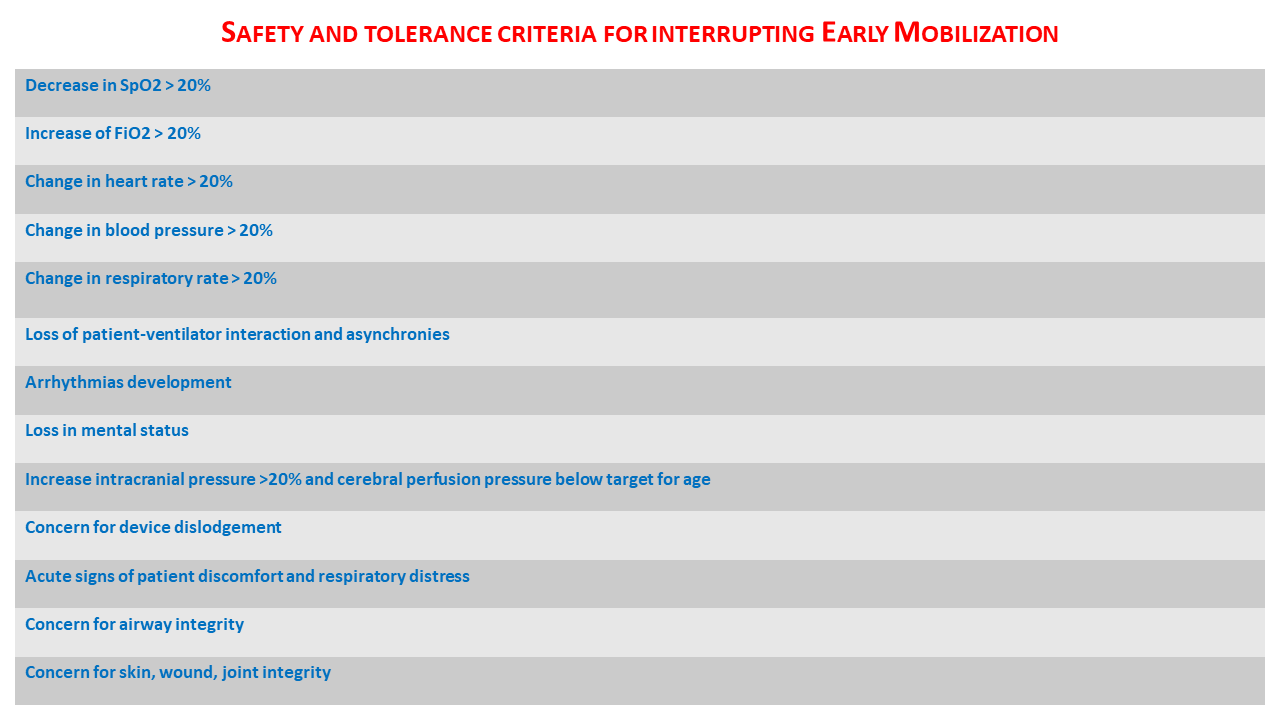


Figure 5 Supplemental material: Safety and tolerance criteria for interrupting mobilization therapy
